# TIM-3 as a molecular switch for tumor escape from innate immunity

**DOI:** 10.3389/fimmu.2012.00418

**Published:** 2013-01-09

**Authors:** Fabrizio Mattei, Giovanna Schiavoni

**Affiliations:** Department of Hematology, Oncology and Molecular Medicine, Istituto Superiore di SanitàRome, Italy

**A commentary on**

**Tumor-infiltrating DCs suppress nucleic acid-mediated innate immune responses through interaction between the receptor TIM-3 and the alarmin HMGB1**

by Chiba et al. (2012). Nat. Immunol. 13, 832–843.

Stimulation of immunity toward tumors has always been one of the most objectives of oncoimmunologists. The identification and characterization of key molecules that may potentially help immunity to contrast cancer progression is increasing over time. Among these factors, TIM-3 is well-recognized to have multiple key roles in eliciting immunity toward pathogens. This transmembrane protein was identified and characterized for the first time 10 years ago by Monney and coworkers, demonstrating the expression of TIM-3 in differentiated Th-1 lymphocytes (Monney et al., 2002). *In vivo*, suppression of TIM-3 was shown to enhance the pathological severity of experimental autoimmune encephalomyelitis (Monney et al., 2002; Anderson and Anderson, 2006).

Here, Chiba and coworkers have evidenced the existence of a comprehensive mechanistic pathway by which the tumor microenvironment affects dendritic cell ability to regulate nucleic acid-mediated innate immune pathways through TIM-3. Indeed, immunosuppressive factors, such as VEGF-A and IL-10 released by tumor cells, induce expression of TIM-3 in dendritic cells which results in impaired response to nucleic acid-stimulated tumor immunity. The authors were able to show that TIM-3 up-regulation occurred selectively in tumor-associated DC (TADC) both in mouse models of transplantable tumors and in tumor samples from patients. Dendritic cells are professional antigen-presenting cells that orchestrate both immune tolerance and immune responses. Through the expression of pattern recognition receptors (PRRs), such as Toll-like receptors (TLRs) and RAGE, which recognize pathogen-associated and damage-associated molecular patterns (PAMPs and DAMPs, respectively) released by stressed cells, DCs play an essential role in initiating innate immune responses against pathogens and tumors. In particular, a number of reports demonstrated that when infiltrating within tumor microenvironment, dendritic cells can contrast tumor progression (Mattei et al., 2009, 2012; Pinto et al., 2012).

The *in vivo* experiments done by Chiba and coworkers show that the therapeutic efficacy of nucleic acid treatment is enhanced when either expression of TIM-3 is suppressed or when DC are depleted. Indeed, injections of antibody to TIM-3 significantly suppress melanoma progression in mice exposed to plasmid DNA or CpG-ODN adjuvants. Furthermore, by using a CD11c-DTR tumor mouse model, where administration of diptheria toxin suppresses dendritic cell lineage, the authors show that selective depletion of DC results in suppression of melanoma growth following treatment with plasmid DNA alone. These data strongly suggest that DC-expressed TIM-3 controls tumor expansion by interfering with the pathways involving Toll-like receptors capable to recognize nucleic acids, such as TLR3, TLR7, and TLR9. Importantly, Type I Interferons and IL-12 produced by conventional and plasmacytoid dendritic cells infiltrating the tumor microenvironment represent a down-stream signal that elicits these DNA/TIM-3-dependent antitumor responses. This assumption is also strengthtened by a large number of reports demonstrating that double-stranded DNA promote the production of Type I Interferons. Indeed, this occurs either *in vitro*, where it was demonstrated that different DC subsets produce this cytokine in response to Poly(I:C), and *in vivo* by intravenous injection of Poly(I:C) in mice (Mattei et al., 2010). Collectively, these observations reveal that Type I Interferons play a significant role in such DNA/TIM-3-controlled antitumor responses.

In their study, Chiba and coworkers also demonstrate the role of the danger/stress signal protein HMGB1 in such TIM-3 dependent pathways. HMGB1 acts not only as transcriptional factor in the nucleus but can be released in the extracellular milieu with effects on inflammation, DC activation and immune responses by binding nucleic acids. The specific receptor for HMGB1 so far recognized is RAGE (Kokkola et al., 2005; Nogueira-Machado et al., 2011). When expressed on dendritic cell surface, this receptor can bind HMGB1, and the function of this RAGE/HMGB1 complex is to allow nucleic acids to activate endosome-driven, TLR-mediated pathways. In their paper, Chiba and coworkers showed that HMGB1 specifically binds to TIM-3 on dendritic cell surface and that this HMGB1/TIM-3 complex is then internalized into endosomal vesicles. This protein complex impedes HMGB-1-mediated recruitment of nucleic acids within the endosomes, leading to a decrease of the efficacy of immune responses to nucleic acids. Indeed, TIM-3 competes with nucleic acids to bind the A box of HMGB1, impeding the HMGB1/DNA association, and thus blocking the activation of innate immune system. Therefore, in the scenario where TIM-3 sequester HMGB1 to nucleic acids, the innate immune responses toward the tumor are blocked resulting in increased tumor expansion.

The authors further extend the implications of their findings in cancer chemotherapy. First, they demonstrate that TIM-3-expressing DC loaded with dying tumor cells promote an immunogenic response (demonstrated by the production of Type I Interferons and IL-12) only when DC were previously treated with a blocking TIM-3 antibody and nucleic acids. In CD11c-DTR tumor-bearing mice, the authors elegantly demonstrate that TIM-3 blockade coupled to administration of cisplatin, an apoptosis-inducing drug (Pruefer et al., 2008), strongly repress melanoma progression, and that TIM-3-expressing DC play a fundamental functional role in this scenario.

The results produced by Chiba and coworkers have important implications in the field of anticancer therapies, as they demonstrated for the first time that TIM-3 functions as an important negative regulator of innate immune responses within the tumor microenvironment. Given the abundant presence of nucleic acids at the tumor site, released by tumor cells dying by constitutive or chemotherapy-induced apoptosis, TIM-3 can be viewed as a new promising target for anticancer therapeutic settings to improve vaccination protocols involving nucleic acids. In addition, these data will provide new insight toward an effective and specific activation of the immunological memory, and potentially opens new perspectives in the field of chemotherapy. To this regard, it will be very interesting to expand and confirm these observations on TIM-3/HMGB1 by employing other chemotherapeutic agents, in order to expect similar effects on suppression of various other solid tumors, in particular those that are surgically not reachable, such as human brain tumors.

Lastly, since TIM-3 exposure on DC surface is a result of tumor-released immunosuppressive factors, which occurs at late stage of tumor development, the data from Chiba and coworkers provide a novel mechanism of tumor escape.
